# Machine learning methods to predict attrition in a population-based cohort of very preterm infants

**DOI:** 10.1038/s41598-022-13946-z

**Published:** 2022-06-22

**Authors:** Raquel Teixeira, Carina Rodrigues, Carla Moreira, Henrique Barros, Rui Camacho

**Affiliations:** 1 EPIUnit – Instituto de Saúde Pública, Universidade do Porto, Rua das Taipas, nº 135, 4050-600 Porto, Portugal; 2 Laboratório para a Investigação Integrativa e Translacional em Saúde Populacional (ITR), Porto, Portugal; 3 CMAT - Centro de Matemática, Universidade do Minho, 4710-057 Braga, Portugal; 4 Departamento de Ciências da Saúde Pública e Forenses e Educação Médica, Faculdade de Medicina, Universidade do Porto, Porto, Portugal; 5 Faculdade de Engenharia da Universidade do Porto, Rua Dr. Roberto Frias, 4200-465 Porto, Portugal; 6 LIAAD-INESC TEC, Rua Dr. Roberto Frias, 4200-465 Porto, Portugal

**Keywords:** Health care, Medical research

## Abstract

The timely identification of cohort participants at higher risk for attrition is important to earlier interventions and efficient use of research resources. Machine learning may have advantages over the conventional approaches to improve discrimination by analysing complex interactions among predictors. We developed predictive models of attrition applying a conventional regression model and different machine learning methods. A total of 542 very preterm (< 32 gestational weeks) infants born in Portugal as part of the European Effective Perinatal Intensive Care in Europe (EPICE) cohort were included. We tested a model with a fixed number of predictors (Baseline) and a second with a dynamic number of variables added from each follow-up (Incremental). Eight classification methods were applied: AdaBoost, Artificial Neural Networks, Functional Trees, J48, J48Consolidated, K-Nearest Neighbours, Random Forest and Logistic Regression. Performance was compared using AUC- PR (Area Under the Curve—Precision Recall), Accuracy, Sensitivity and F-measure. Attrition at the four follow-ups were, respectively: 16%, 25%, 13% and 17%. Both models demonstrated good predictive performance, AUC-PR ranging between 69 and 94.1 in Baseline and from 72.5 to 97.1 in Incremental model. Of the whole set of methods, Random Forest presented the best performance at all follow-ups [AUC-PR_1_: 94.1 (2.0); AUC-PR_2_: 91.2 (1.2); AUC-PR_3_: 97.1 (1.0); AUC-PR_4_: 96.5 (1.7)]. Logistic Regression performed well below Random Forest. The top-ranked predictors were common for both models in all follow-ups: birthweight, gestational age, maternal age, and length of hospital stay. Random Forest presented the highest capacity for prediction and provided interpretable predictors. Researchers involved in cohorts can benefit from our robust models to prepare for and prevent loss to follow-up by directing efforts toward individuals at higher risk.

## Introduction

Attrition, the loss of participants belonging to the initial sample of recruitment who do not return for subsequent follow-ups, is one of the most challenging problems faced by researchers in charge of cohorts^1^. Importantly, a cohort affected with attrition may have the validity of its results questioned, as attrition introduces selection bias if related to the outcome of interest^2,3^.

Efforts to tackle attrition in cohorts have been concentrated in two main actions: prevent its occurrence and develop statistical methods to alleviate its consequences in data analysis^1^. For the latter, regression imputation, inverse probability weighting, and multiple imputation are some of the available techniques^4–6^. To prevent or diminish the loss of participants during the study, retention strategies have been widely implemented, such as voucher incentives, reminders, birthday cards, and reimbursement of transport costs^7^. However, conflictual results on the effectiveness of these strategies^7,8^ suggest that there may not be a unique solution for all types of cohorts, settings, and participants, but rather specifically tailored strategies are required.

Birth cohorts of high-risk children, like those born very preterm (< 32 weeks of gestation), have an important role in providing a comprehensive assessment of the needs and development of these children across their lifespan^9^. Very preterm infants experience increased and long-term adverse outcomes, such as cognitive and behavioural problems, when compared with children born at term^10^. Hence, this type of cohort may provide valuable scientific evidence that, ultimately, will contribute to improving clinical care, supporting public health decisions, and planning health and education provisions to these children^11^.

An early and precise identification of which participants present an increased risk for dropping out may be of large benefit. Conventional statistical methods, such as Logistic Regression, have been the usual choice to predict attrition in cohorts^12–14^. However, these classical theory-based models are constrained by independence, additivity and linearity assumptions which may oversimplify complex relationships between predictors and outcome variables^15^.

The growing access to clinical data and the rapid advances in machine learning raised a great enthusiasm about its use to improve clinical care over the past decade^16^ and an increasing number of its application in epidemiological research and practice is known^17^. In addition, machine learning methods may bring advantages over conventional approaches. It offers highly flexible algorithms that often do not require underlying distributional assumptions or model specification, and is able to adapt to complex non-linear and non-additive interrelations between outcome and covariates^18^. However, when it concerns employing machine learning techniques to address methodological challenges in epidemiological studies, the results are scarce.

In this study, we developed predictive models of attrition in a birth cohort of very preterm infants applying a conventional regression model and different machine learning methods, and looked for the most relevant predictors of attrition.

## Methods

### Study population

The study population consisted of Portuguese children participating in the prospective population-based Effective Perinatal Intensive Care in Europe (EPICE) cohort. It included all very preterm births (between 22 + 0 and 31 + 6 weeks of gestation) in 2011/12 in 19 regions of 11 European countries^19^. In Portugal, there were 724 very preterm live births occurring in this period in the two geographic regions (Northern and Lisbon and Tagus Valley) included in the cohort^20^. This study included all infants discharged alive from Neonatal Intensive Care Units (NICUs) whose parents provided written informed consent to participate in the EPICE cohort in Portugal (EPICE-PT) and to be long-term followed-up, resulting in 544 children (89.6% of 607 eligible participants)^19^. We excluded two infants who died after discharge, remaining 542 participants for the analysis. Participant’s data at baseline were extracted from medical charts by health care professionals using a pretested standardized questionnaire^19^. In this study, we focused on the first four years of follow-up (follow-up 1–follow-up 4), where questionnaires on child's health and development were administered to parents by telephone (follow-up 1, 3 and 4) and postal questionnaires (follow-up 2).

### Outcome

The outcome of interest was attrition, i.e., non-participation in offered follow-ups. Attrition was identified when the participant (a) could not be reached by any available contact (including relative’s contact), (b) repeatedly postponed the call to answer the questionnaire, (c) verbally refused to participate in that specific follow-up, (d) verbally requested to withdrawal from the cohort, or (d) did not mail the questionnaires back, even after several reminders (follow-up 2). Attrition at each follow-up was calculated considering the eligible participants, i.e., excluding possible deaths and/or previous formal refusals. Participation was considered when parents accepted the invitation for that specific follow-up and answered the questionnaires (either totally or partially) through any available method.

### Predictors

Predictors were taken from information collected at baseline and from questionnaires completed at the three subsequent follow-ups. Based on the literature and experience of the researchers involved in the cohort, we selected a list of demographic, socioeconomic and clinical characteristics that are likely to be important predictors of attrition (Supplementary Table 1). The decision to not include all predictors available in the cohort dataset was taken to mitigate the curse of dimensionality^21^, to diminish the computational costs, prevent overfitting^22^ and, increase the usability of the model in similar cohorts.

### Model development

Two predictive models framework were developed: (1) “Baseline”, where prediction of the first four follow-ups was done using baseline data only, independently and, (2) “Incremental”, where baseline variables were used to predict attrition in the follow-up 1 and from that on, we continuously added new predictors extracted from the subsequent follow-up (e.g. baseline plus follow-up 1 to predict attrition in the follow-up 2; baseline plus follow-up 1 and 2 to predict attrition in the follow-up 3, etc.). For the first follow-up, both models are equivalent.

To test the model's performance in predicting new data, we have used, for each year, 5 repetitions with replacement of a hold-out method^23^. In each of the five folds, the whole dataset was randomly split into a training set (80%) and a testing (20%). Most machine learning algorithms have a set of parameters that may be adjusted to get a good model (parameter tunning). We have adopted a wrapper approach^24^ to estimate the best combination of parameter’s values. We have split the training set into a tuning-training set (95% of the original training set) and a tuning-test set (5% of the original training set). The result of the wrapper is the parameter’s values that produced the best (AUC-PR) value on the prune-test set. The best combination of parameter values is used on the training set and the model is finally evaluated on the test set.

The prevalence of the outcome (attrition) in the various follow-up of EPICE-PT cohort ranged from 13 to 25%. Hence, we have a set of imbalanced datasets, which turns the models prone to be biased towards the majority class. In order to cope with this problem, the Synthetic Minority Over-Sampling Technique (SMOTE)^25^ was applied to mitigate the imbalance of the datasets.

### Classification methods

Different classification methods were leveraged to build the predictive models. Selected machine learning methods included AdaBoost, Artificial Neural Networks, K-Nearest Neighbours, Decision Trees Classifiers (Functional Trees, J48 and J48Consolidated), and Random Forest. We also applied Logistic Regression, performed with identical predictors, without interaction terms. A short explanation of the different methods is described below:

*AdaBoost* is one of the most popular boosting algorithms, a group of methods that produce a classifier as a linear combination of weak classifiers, and does so in a way that minimizes exponential loss over such linear combinations^26^. A weak classifier can be described as one whose error rate is only slightly better than random guessing^15^.

*Artificial Neural Networks* are nonlinear statistical models, which extract linear combinations of the predictors as derived features, and then generate an outcome as a nonlinear function of these features. This learning method, inspired by neuroscience, is quite robust to noise in the training data^15,27^.

*K-Nearest Neighbours* models are based on the sample’s geographic neighbourhood. It uses the nearest observations, based on a distance measure, to predict the final classification outcome of a new observation^28^.

*Decision Trees Classifiers (Functional Trees, J48 and J48Consolidated)* are a group of algorithms that use a binary recursive partitioning of instant space^29^. It is an iterative process of splitting the data into partitions, and then splitting it up further on each of the branches, aiming to partition the data into smaller, more homogeneous groups. By fully revealing the feature space partition of a single tree, it allows for great flexibility in data analysis and interpretability^15,29^.

*Random Forest* algorithms are an extension of bagging^30^, an ensemble learning method that builds successive independent trees using a bootstrap sample of the data set. It adds a new layer of randomness when selecting predictors or combinations of predictors at each node to split it, while bagging considers all of the original predictors for splitting a node^31^.

*Logistic Regression* is typically the foremost statistical analysis used to model binary responses. It belongs to a family of techniques called Generalized Linear Models, which models the log odds of a binary dependent variable as a linear function^28^.

All models and algorithms were run using WEKA^32^.

### Performance metrics

We used four metrics to estimate the performance of the different classification methods^33^: (1) Sensitivity: the ability of the model to identify all the relevant cases (dropouts) within the dataset, (2) Accuracy: it measures the fraction of all correct predictions, (3) F- measure: conveys the balance between precision and sensitivity and (4) AUC-PR: Area Under the Curve of Precision-Recall. AUC-PR was the primary metric adopted to assess the performance of the algorithms, given the purpose of our study is to identify the cohort’s participants more prone to attrition and to select a predictive model that is as generalizable as possible to other cohorts of very preterm infants.

### Predictor variables importance

We collected the variable rank given by the best algorithm in each run and then we calculated the overall mean rank of the five best variables over all runs. To investigate the effects of the most relevant continuous predictor variables across different values, partial dependence plots were generated for the most accurate algorithm^34^. Aiming to improve interpretability, partial dependence plots were stratified by categories, when appropriated. The plots were presented with smooth curves to allow possible important patterns to more clearly stand out. Graphs were constructed using R programming language.

### Ethics

The EPICE-PT cohort was approved by the Ethics Committee of the participating hospitals and by the Portuguese Data Protection Authority (authorization 7426/2011)^20^. All research was performed in accordance with relevant guidelines and informed consent was obtained from all parents or legal representatives, as required by national legislation. The study complies with the Helsinki Declaration 2008.

### Ethics committees that approved the study

1. Ethics Committee of Hospital Center Alto Ave—Guimarães
2. Ethics Committee of Hospital Center Entre Douro e Vouga—Hospital São Sebastião
3. Ethics Committee of Hospital Center Médio Ave—Hospital de Famalicão
4. Ethics Committee of Hospital Center Porto—Maternidade Júlio Dinis
5. Ethics Committee of Hospital Center Póvoa de Varzim /Vila do Conde—Hospital Póvoa Varzim
6. Ethics Committee of Hospital Center São João—Hospital São João
7. Ethics Committee of Hospital Center Tâmega e Sousa—Hospital Padre Américo
8. Ethics Committee of Hospital Center Trás dos Montes e Alto Douro—Hospital São Pedro
9. Ethics Committee of Hospital Center Vila Nova de Gaia/Espinho—Unidade II
10. Ethics Committee of Hospital São Marcos—Hospital São Marcos
11. Ethics Committee of Local Health Unit Matosinhos—Hospital Pedro Hispano
12. Ethics Committee of Local Health Unit Alto Minho—Hospital de Santa Luzia
13. Ethics Committee of Hospital Center Nordeste—Hospital Bragança
14. Ethics Committee of Hospital Center de Setúbal—Hospital São Bernardo
15. Ethics Committee of Hospital Center Barreiro/Montijo—Hospital São Bernardo ~ 
16. Ethics Committee of Hospital Center Oeste—Hospital das Caldas da Rainha
17. Ethics Committee of Hospital Center Oeste—Hospital de Torres Vedras
18. Ethics Committee of Hospital Center Lisboa Central—Hospital Dona Estefânia
19. Ethics Committee of Hospital Center Lisboa Central—Maternidade Alfredo da Costa
20. Ethics Committee of Hospital Center Lisboa Norte—Hospital de Santa Maria
21. Ethics Committee of Hospital Center Lisboa Ocidental—Hospital de São Francisco de Xavier
22. Ethics Committee of Hospital Center Médio Tejo—Hospital de Abrantes
23. Ethics Committee of Hospital CUF Descobertas
24. Ethics Committee of Hospital Fernando Fonseca
25. Ethics Committee of Hospital da Luz
26. Ethics Committee of Hospital de Santarém
27. Ethics Committee of Hospital de Vila Franca de Xira
28. Ethics Committee of Hospital dos Lusíadas
29. Ethics Committee of Hospital Garcia de Horta
30. Ethics Committee of Hospital José de Almeida

## Results

Of the 542 very preterm children included in the study, 57.2% were male. The median gestational age was 29 weeks (p25–p75:27–31) and the median birthweight was 1172 g (p25–p75: 940–1436.2). Mothers were mostly primiparous (63.2%), native (84.9%), with a median age of 31 years (p25–p75:27–35) and 83.2% belonged to the least deprived quartiles of neighbourhood socioeconomic deprivation (Table 1). Attrition in the four follow-ups were, respectively: 16%, 25%, 13% and 17%.

**Table 1:** General characteristics of the study population (n = 542).

Characteristics	n^a^ (%)
**Sex**	
Female	232 (42.8)
Male	310 (57.2)
**Birthweight (g)**	
Median (p25–p75)	1172 (940–1436)
**Gestational age (weeks)**	
Median (p25–p75)	29 (27–31)
< 26	27 (5.0)
26–27	118 (21.8)
28–29	148 (27.3)
30–31	249 (45.9)
**Small for gestational age**^**b**^	
Yes (< 10th percentile)	52 (9.7)
No (≥ 10th percentile)	485 (90.3)
Missing	5 (0.9)
**Type of pregnancy**	
Singleton	372 (68.6)
Multiple	170 (31.4)
**Parity**	
0	342 (63.2)
1	144 (26.6)
≥ 2	55 (10.2)
Missing	1 (0.2)
**Caesarean**	
No	156 (29.1)
Yes	381 (70.9)
Missing	5 (0.9)
**Maternal age**^**c**^	
Median (p25–p75)	31 (27–35)
< 25	85 (15.7)
25–34	300 (55.4)
≥ 35	157 (29.0)
**Native mother**	
No	81 (15.1)
Yes	454 (84.9)
Missing	7 (1.3)
**Neighborhood socio-economic deprivation**	
Least deprived (q1–q4)	447 (83.2)
Most deprived (q5)	90 (16.8)
Missing	5(0.9)
**Length of hospital stay (days)**	
Median (p25–p75)	51(37–71)

^a^Calculation of percentages does not include missing values.

^b^SGA, small for gestational age, based on intrauterine curves developed for the cohort^54^.

^c^The sum of the categories surpasses 100% as the numbers were rounded up.

The SMOTE technique improved the performance of all algorithms in both models, therefore, all the presented results are derived using this technique. To verify the reliability of the results with the oversampling technique, we compared the descriptive statistics of the original dataset and the oversampling counterpart and we found no significant differences.

### Comparison of methods performance

**Figure 1:**
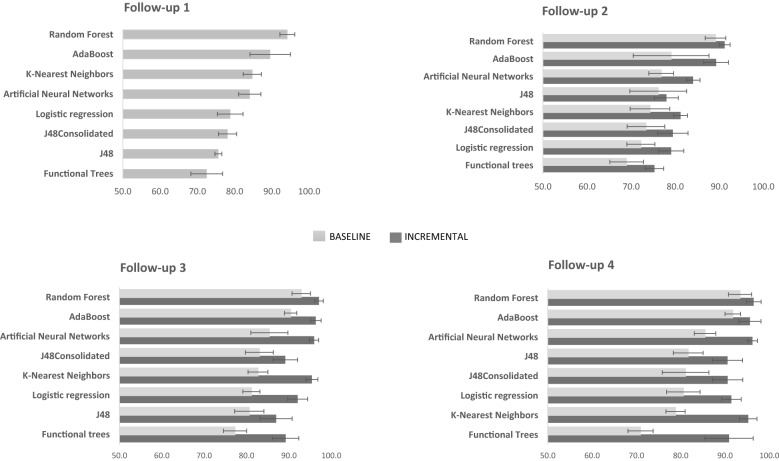
Area Under the Curve-Precision Recall (AUC-PR) for follow-ups 1, 2, 3 and 4.

Figure 1 depicts the discriminatory abilities of all methods for the prediction of attrition. There was a consistent and large superiority of Random Forest over the other methods in the baseline model. For the incremental one, Random Forest also had the best performance, but only slightly higher than AdaBoost (follow-up 2, 3 and 4) and Artificial Neural Networks (follow-3 and 4). Discrimination performance of Random Forest was excellent across all follow-ups in both models, baseline [AUC-PR_1_: 94.1 (2.0); AUC-PR_2_: 89.1 (2.3); AUC-PR_3_: 92.9 (2.2); AUC-PR_4_: 93.4 (2.6)] and incremental [AUC-PR_1_: 94.1 (2.0); AUC-PR_2_: 91.2 (1.2); AUC-PR_3_: 97.1(1.0); AUC-PR_4_: 96.5 (1.7)]. In all follow-ups, the conventional Logistic Regression approach had a worse performance than Random Forest, both in baseline [AUC-PR_1_: 78.8 (3.4); AUC-PR_2_: 72.2 (3.2); AUC-PR_3_: 81.1(2.0); AUC-PR_4_: 80.6 (3.8)] and incremental model [AUC-PR_1_: 78.8 (3.4); AUC-PR_2_: 79.1 (2.9); AUC-PR_3_: 92.1 (2.3); AUC-PR_4_: 91.4 (2.2)]. Supplementary Table 2 presents the odds-ratios of the Logistic Regression for the most relevant predictors. Adding new predictors in the incremental model led to a greater performance of all algorithms in all follow-ups.

Table 2 presents the mean and standard deviation of the assessed metrics (sensitivity, accuracy and F-measure). At follow-up 1, Random Forest (82.3; 6.3) and AdaBoost (82.3; 6.0) presented the higher values for sensitivity, which measures the proportion of positive cases (dropouts) that were correctly identified. At follow-up 2, K-Nearest Neighbours (87.6; 4.5) at the baseline model outperformed the other methods. Random Forest was the best algorithm for sensitivity in follow-3 (89.8; 4.1) and Functional Trees in follow-up 4 (91.5; 3.7), both at the incremental model. In an overall analysis of the three metrics, Random Forest presented the best performance in both models, at all follow-ups.

**Table 2:** Performance results of the classification methods applied to the prediction of attrition in four follow-ups of EPICE-PT cohort.

Follow-up	Methods	Performance metrics (mean, SD)											
		Baseline model						Incremental model^a^					
		Sensitivity		Accuracy		F-measure		Sensitivity		Accuracy		F-measure	
1	AdaBoost	82.3	6.0	83.2	5.7	83.3	5.7	N/a	N/a	N/a	N/a	N/a	N/a
	Artificial Neural Networks	81.4	3.1	81.1	3.1	81.2	3.1	N/a	N/a	N/a	N/a	N/a	N/a
	Functional Trees	74.5	5.2	74.7	1.8	74.7	1.8	N/a	N/a	N/a	N/a	N/a	N/a
	J48	76.9	3.3	78.0	2.9	78.0	2.8	N/a	N/a	N/a	N/a	N/a	N/a
	J48Consolidated	82.0	4.2	79.3	2.0	79.3	1.9	N/a	N/a	N/a	N/a	N/a	N/a
	K-Nearest Neighbours	86.0	3.9	76.5	2.1	76.5	2.2	N/a	N/a	N/a	N/a	N/a	N/a
	Logistic Regression	69.7	5.7	73.7	2.0	73.6	2.1	N/a	N/a	N/a	N/a	N/a	N/a
	Random Forest	82.3	6.3	88.2	1.9	88.1	2.0	N/a	N/a	N/a	N/a	N/a	N/a
2	AdaBoost	82.4	5.8	71.6	7.2	70.9	7.6	85.6	3.6	82.3	3.7	82.3	3.7
	Artificial Neural Networks	82.6	6.3	75.2	3.5	74.8	3.5	82.2	1.8	79.9	1.9	79.9	2.0
	Functional Trees	76.8	3.8	71.4	2.6	71.2	2.6	76.1	2.8	73.1	3.2	73.1	3.2
	J48	77.8	7.4	73.2	5.3	73.1	5.3	79.4	3.1	77.0	1.8	76.9	1.9
	J48Consolidated	73.7	4.1	73.6	4.2	73.6	4.3	76.5	4.1	78.1	1.5	78.2	1.5
	K-Nearest Neighbours	87.6	4.5	71.7	3.9	70.5	4.0	85.4	2.7	76.7	1.6	76.4	1.7
	Logistic Regression	77.2	2.5	67.0	1.7	66.4	1.8	80.2	4.7	74.7	2.5	74.6	2.4
	Random Forest	86.8	2.4	82.6	1.8	82.5	1.8	85.0	3.3	84.6	2.5	84.6	2.5
3	AdaBoost	75.4	6.2	85.0	3.5	84.8	3.6	87.9	7.3	90.3	1.7	90.3	1.8
	Artificial Neural Networks	79.0	7.0	81.3	3.1	81.3	3.2	87.2	5.1	89.8	0.3	89.8	0.3
	Functional Trees	74.4	5.7	78.2	3.0	78.3	3.0	84.9	6.0	87.5	2.1	87.5	2.1
	J48	70.8	3.4	81.0	2.2	80.8	2.2	84.2	6.4	89.0	2.7	89.0	2.8
	J48Consolidated	74.1	4.6	80.5	2.7	80.5	2.7	87.8	3.0	89.6	1.9	89.6	1.9
	K-Nearest Neighbours	72.5	2.6	77.7	2.0	77.7	1.9	88.9	6.6	90.1	1.8	90.1	1.9
	Logistic Regression	69.5	5.5	77.6	1.1	77.4	1.2	87.9	6.4	88.1	3.0	88.2	3.1
	Random Forest	73.4	3.8	86.1	2.1	85.7	2.2	89.8	4.1	92.9	0.9	92.9	0.9
4	AdaBoost	83.3	3.1	84.2	1.5	84.2	1.5	88.5	4.5	92.1	2.6	92.1	2.6
	Artificial Neural Networks	82.3	4.0	78.4	2.9	78.4	2.9	91.0	1.6	92.9	2.1	92.9	2.1
	Functional Trees	76.2	4.1	74.3	1.2	74.2	1.2	91.5	3.7	92.2	3.1	92.2	3.1
	J48	74.6	5.6	79.6	2.5	79.5	2.6	88.7	3.4	92.5	1.7	92.4	1.7
	J48Consolidated	77.4	4.3	77.0	5.4	77.0	5.3	89.2	3.3	92.7	1.6	92.7	1.6
	K-Nearest Neighbours	84.1	1.0	72.6	2.0	72.4	2.1	89.0	1.5	93.3	1.4	93.3	1.4
	Logistic Regression	76.1	3.0	73.5	1.8	73.6	1.9	87.7	4.9	89.2	1.6	89.2	1.6
	Random Forest	82.6	3.0	85.3	2.3	85.2	2.3	91.0	2.3	94.3	2.2	94.2	2.2

^a^At follow-up 1, baseline and incremental model are equivalent.

### Predictor importance analysis

**Figure 2:**
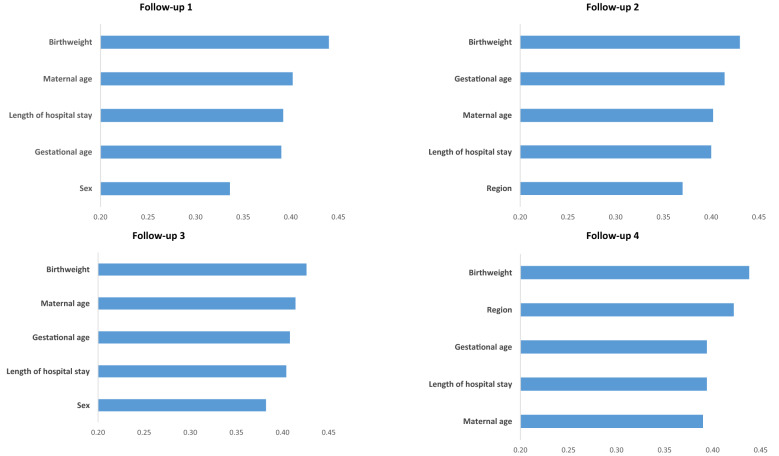
Importance of the predictor variables (based on the mean decrease in impurity) in the Random Forest for each year (Baseline Model).

Predictor importance was computed by evaluating the decrease of impurity at each split across all decision trees in the forest^35^. Either in baseline or incremental model, of the five most relevant predictors, four were common for all follow-ups and circumscribed to clinical and demographic characteristics: birthweight, gestational age, maternal age, and length of hospital stay after birth. Region of birth (Lisbon and Tagus Valley) and sex of the child (male) were the other two more relevant predictors (Table 3). Figure 2 shows the top five predictors with the highest importance based on the Random Forest in Baseline model.

**Table 3:** The top- ranked variables by the variable importance for each year in Baseline and Incremental Model.

Mean rank	Follow-up 1	Follow-up 2	Follow-up 3	Follow-up 4
**Baseline**				
1	Birthweight	Birthweight	Birthweight	Birthweight
2	Maternal age	Gestational age	Maternal age	Region of birth
3	Length of hospital stay	Maternal age	Gestational age	Gestational age
4	Gestational age	Length of hospital stay	Length of hospital stay	Length of hospital stay
5	Sex	Region of birth	Sex	Maternal age
**Incremental**				
1	Birthweight	Birthweight	Birthweight	Birthweight
2	Maternal age	Maternal age	Length of hospital stay	Maternal age
3	Length of hospital stay	Gestational age	Gestational age	Gestational age
4	Gestational age	Sex	Sex	Region of birth
5	Sex	Length of hospital stay	Maternal age	Length of hospital stay

Partial dependence plots illustrating the effects of the continuous predictors across a range of values in the Random Forest algorithm are shown in Supplementary Figs. 1, 2, 3 and 4. As the plots are similar for baseline and incremental models, we opted to display only the baseline model. The risk for attrition increased with higher gestational age and lower maternal age, although the risk also increases for older mothers (> 35 years) at follow-ups 3 and 4. The stratification of birthweight by sex revealed different tendencies. For male participants, the risk for attrition has an inverted U-shape, with a lower risk for extreme values; and it shows two peaks of increased risk (1000 and 2000 g) for females. Length of hospital stay after birth was stratified by gestational age (≤ 27 and > 27 weeks). In both categories, the risk increased with length of hospital stay, with a more rapid increase generally occurring after 50 days.

## Discussion

Using seven machine learning algorithms and conventional Logistic Regression, this study developed two models for characterizing the risk of attrition in the EPICE-PT cohort. Both models presented an optimal predictive performance, with the best performance reached by the incremental one, in which new predictors were progressively added. The Random Forest showed the best discrimination performance in all follow-ups, surpassing Logistic Regression. In addition, we achieved a good level of interpretability of the predictors, emphasizing the added value of this algorithm. Random Forest not only improved the discriminative ability but also provided clear information for supporting the development of tailored retention strategies along the cohort life cycle. Based on the results of the Random Forest algorithm, younger mothers, children born with higher gestational age and with longer length of hospital stay presented more risk of dropping out. Birthweight, sex, and region of birth were also among the most important risk factors for attrition.

The two predictive models of attrition have distinct advantages. The baseline model resulted in an excellent predictive performance, also offering the opportunity to predict attrition and plan tailored interventions to prevent it at an early stage of the cohort. The incremental model achieved an even higher predictive performance compared to the baseline model and improves the performance of the other algorithms, broadening the option of satisfactory methods. However, it increases the computational costs, is more time-consuming and less efficient at identifying potential dropouts at an early stage, which is a substantial disadvantage from the perspective of cohort maintenance. In both models, all the top-ranked predictors belonged to the baseline dataset. For these reasons, we consider the baseline model the most advantageous one to predict attrition in our study population and similar cohorts.

A superior performance of Random Forest over Logistic Regression for predictive models was shown in diverse biomedical applications, such as suicidal behaviour^36^, cancer metastasis^37^, readmissions in patients with heart failure^38^ and, unplanned rehospitalisation of preterm babies^39^. Likewise, a massive experimental evaluation of 179 algorithms using 121 datasets showed that Random Forest was very close to the best attainable accuracy for most of the datasets^40^. However, a systematic review consisting of 71 studies did not favoured machine learning methods over Logistic Regression for clinical prediction^41^. These discrepant results may be explained by the No-Free-Lunch theorem^42^, which states that no classifier can be always the best for all datasets. Nevertheless, the comparison of our model’s performance with previous research is limited by the lack of studies investigating the ability of machine learning methods to predict attrition in cohorts.

Identifying the key predictors of attrition is of great significance for mitigating its risk in cohorts. Although the top-ranked predictors of attrition in our research are non-modifiable variables, they certainly shed light on which participants should receive further attention and incentives to continue their participation. The identified predictors are consistent with previous findings in very preterm cohorts, such as lower maternal age^43,44^ and male sex^45,46^. The effects of the most relevant clinical predictors showed controversial results, either revealing that participants with better (higher gestational age, greater birthweight in females, average birthweight in males) or worse health (longer length of hospitalisation) are more prone to attrition. A systematic review of 57 publications of very preterm cohorts also identified the healthier (e.g., higher gestational age, better lung function) and the unhealthier participants (e.g., severe disabilities, poorer cognitive performance), more likely to drop out of the cohort^47^. Therefore, this paradox is not a new finding and remains to be elucidated. It is also important to refer to the noticeable absence of socioeconomic factors in our model, which are often among the strongest predictors of attrition^43,44,48^. This might be due to the small variability of our sample regarding the only socioeconomic indicator among our baseline predictors, neighbourhood socioeconomic deprivation index^49^ (82.5% of the participants belong to the least deprived quartiles).

Our study’s strengths include: (1) data from a population-based prospective cohort, which represented almost 70% of all VPT births that occurred in Portugal in 2011/2012, (2) several machine learning methods tested, given that the most appropriate algorithm may differ depending on data structure, (3) the selection of usual predictors collected at very preterm cohorts instead of all available predictors in our dataset, to broaden the usability of the model for similar cohorts, (4) the satisfactory level of model interpretation, allowing further practical implementation of the obtained results. Moreover, to the best of our knowledge, this is the first study developing prediction models of attrition in longitudinal cohort studies through machine learning techniques.

The primary limitation of the current study is that we assessed the performance of machine learning models by the hold-out method, a form of internal validation. External validation in other very preterm cohorts is needed to confirm the performance of the developed models. Another limitation was the lack of information on sociodemographic indicators at baseline, important known predictors of attrition, such as mother’s employment^50^ and educational level^51^. Though the availability of such information at baseline would likely improve the prediction ability, our models performed well enough. Moreover, the neighbourhood socioeconomic deprivation index is a robust measure that has been used as a valid proxy of individual socioeconomic position in previous research^52^. Lastly, variable importance of Random Forest was estimated by the mean decrease in impurity (or Gini importance) mechanism, which may produce biased variable selection when predictor variables vary in their scale of measurement or number of categories, such as in our dataset. Notwithstanding, the identified top-ranked predictors are in line with previous research on attrition in very preterm cohorts, reassuring our results. In addition, previous research has demonstrated that when Random Forest uses a significant number of trees in each run, which is our case, stable variable importance rankings are achieved^53^.

In conclusion, we have developed and validated robust machine learning predictive models of attrition in a cohort of very preterm infants and demonstrated their superiority and feasibility compared with conventional Logistic Regression. Other than the high-performance model, this study also provided interpretability of the most relevant predictors that contribute to attrition. Researchers involved in cohorts lack effective tools to early identify participants at risk of attrition and can benefit from our results to prepare for and prevent loss to follow-up, e.g., by directing efforts and developing tailored interventions geared toward those individuals to promote their continued participation^54–56^.

## Supplementary Information

Supplementary Information 1.

Supplementary Information 2.

Supplementary Information 3.

## Data Availability

Participants data used for modelling are available to researchers upon reasonable request.
