# Supplementary material for: Machine learning methods to predict attrition in a population-based cohort of very preterm infants
Source: Sci Rep. 2022 Jun 22;12:10587. doi: 10.1038/s41598-022-13946-z (PMC9217966; doi:10.1038/s41598-022-13946-z)
Supplement: Supplementary file 1 — Supplementary Information 1. [file 41598_2022_13946_MOESM1_ESM.docx]

Supplementary Figure 1. Partial dependence plots on effect of birthweight on the risk of attrition, stratified by sex: female (a) and male (b).

Follow-up 1 Follow-up 2 Follow-up 3 Follow-up 4
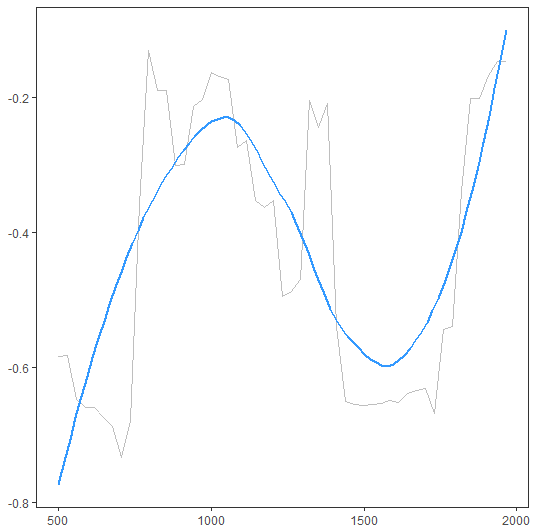

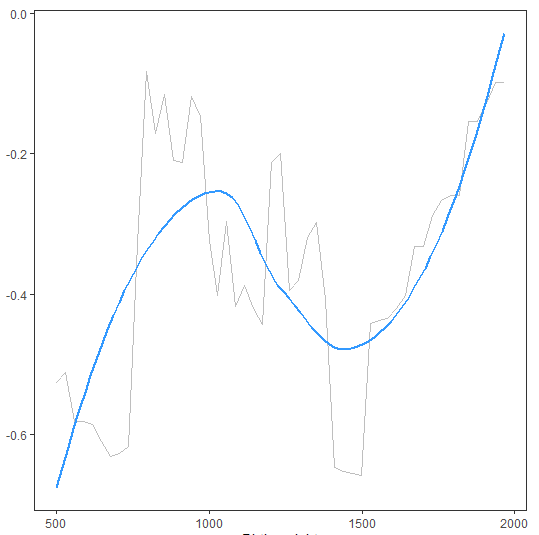

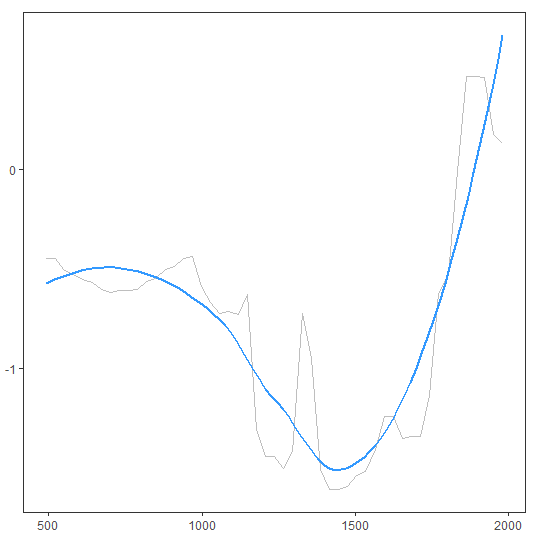

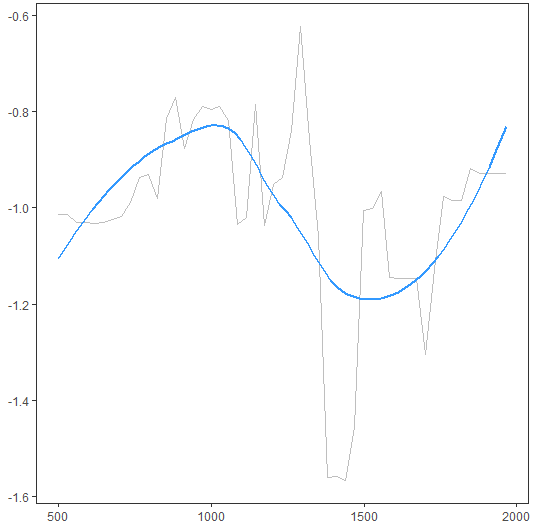


(a)

Partial Dependence


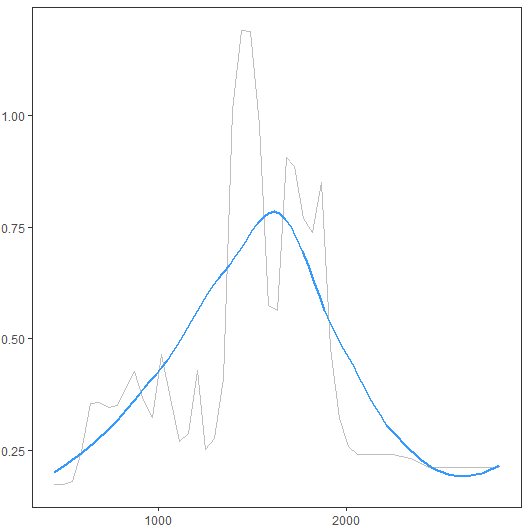

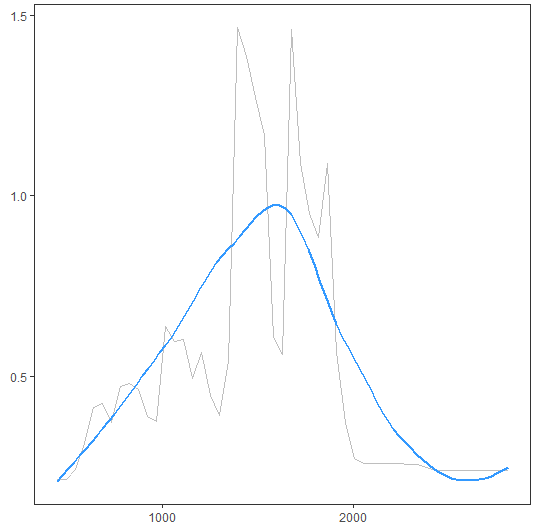

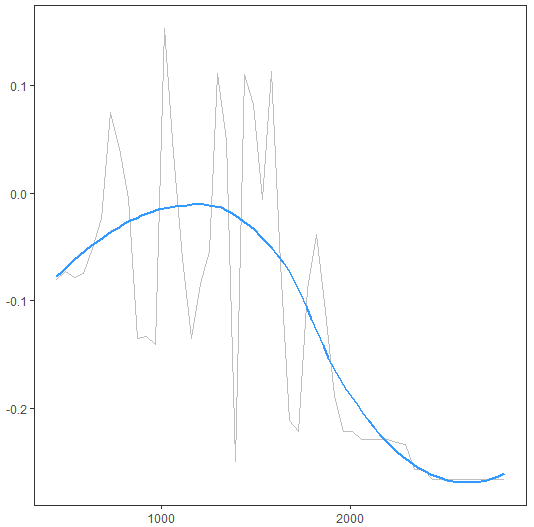

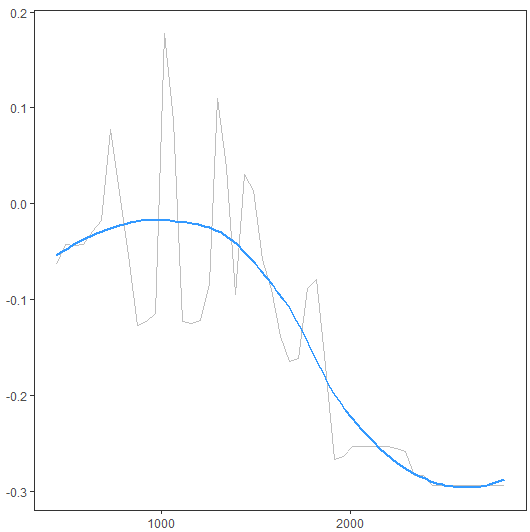


(b)

Birthweight Birthweight Birthweight Birthweight

Supplementary Figure 2. Partial dependence plots on effect of gestational age on the risk of attrition.

Follow-up 1 Follow-up 2 Follow-up 3 Follow-up 4
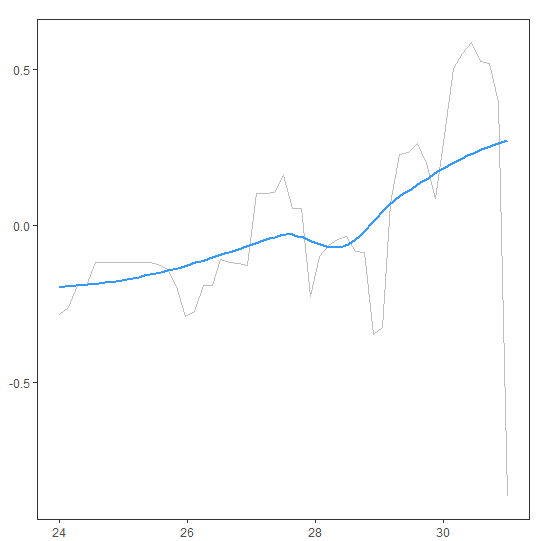

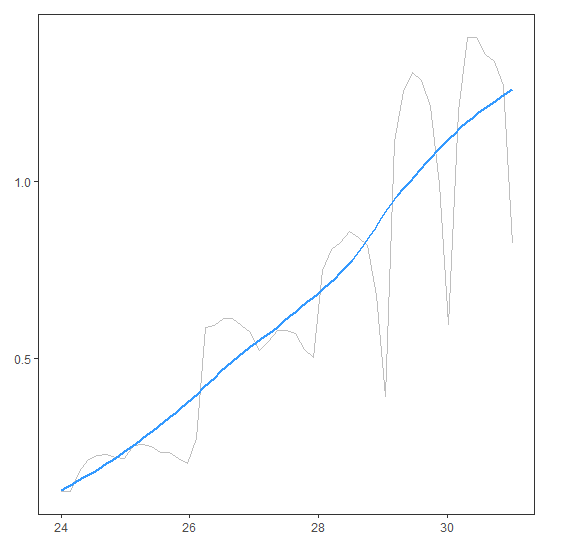

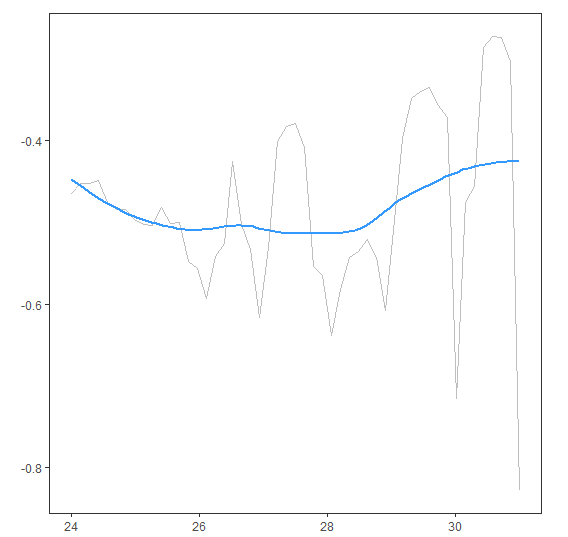

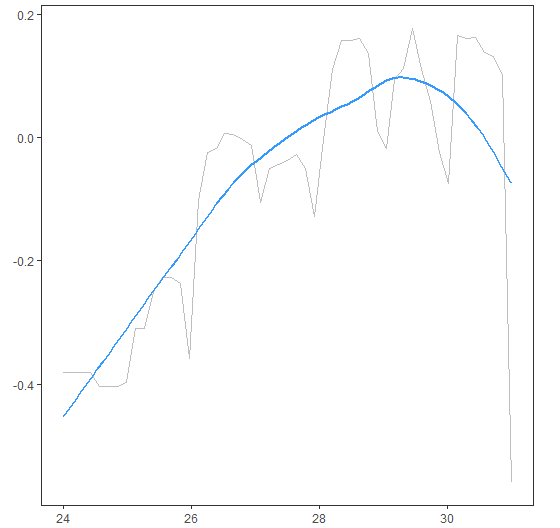


Partial Dependence

Gestational age Gestational age Gestational age Gestational age

Supplementary Figure 3. Partial dependence plots on effect of maternal age on the risk of attrition.

Follow-up 1 Follow-up 2 Follow-up 3 Follow-up 4


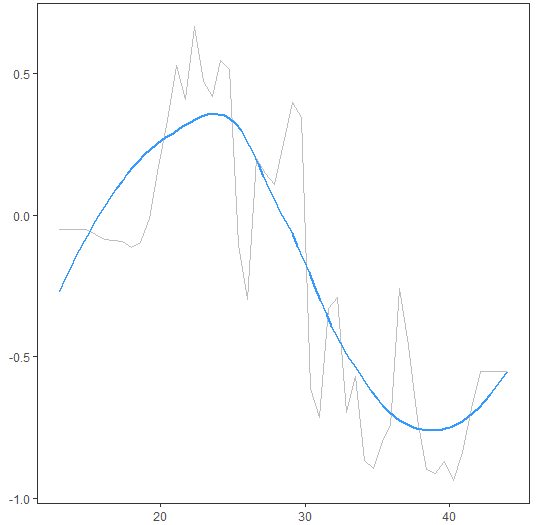

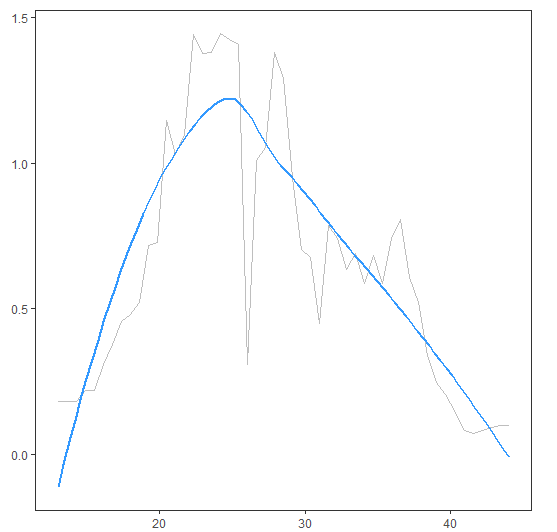

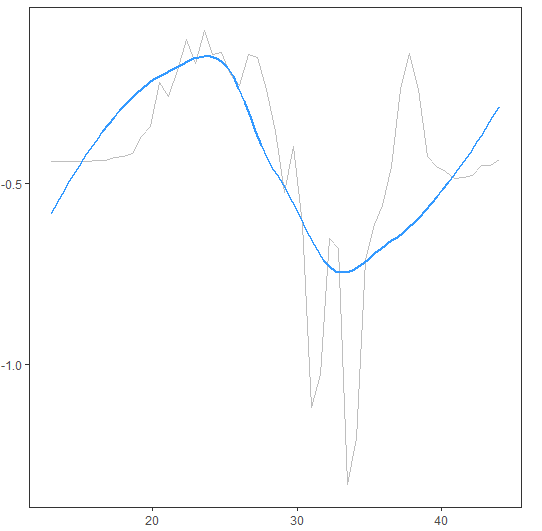

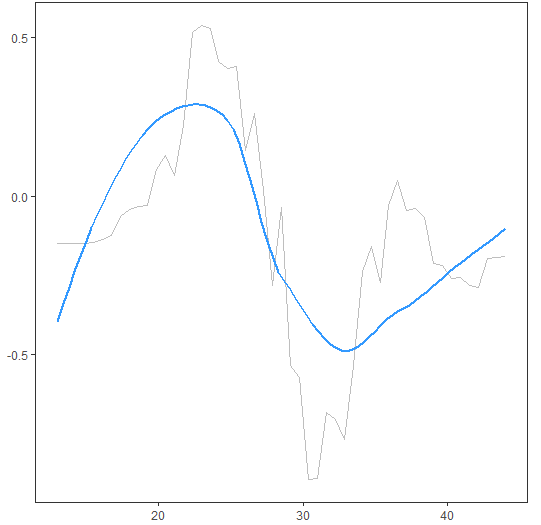
 Maternal age Maternal age Maternal age Maternal age

Partial Dependence

Supplementary Figure 4. Partial dependence plots on effect of length of hospital stay on the risk of attrition, stratified by gestational age: (a) ≤ 27 and (b) > 27 weeks.

Follow-up 1 Follow-up 2 Follow-up 3 Follow-up 4
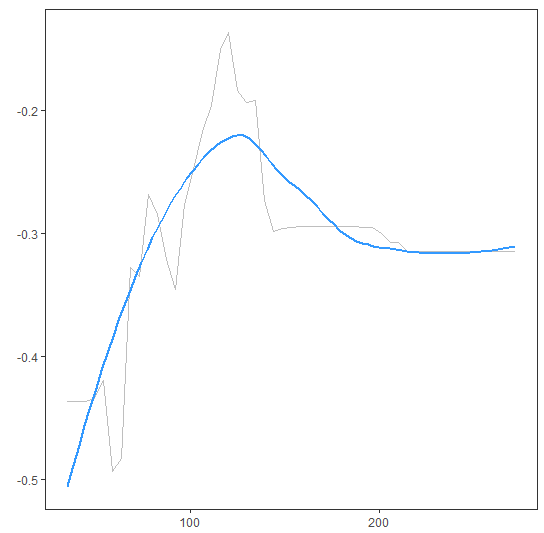

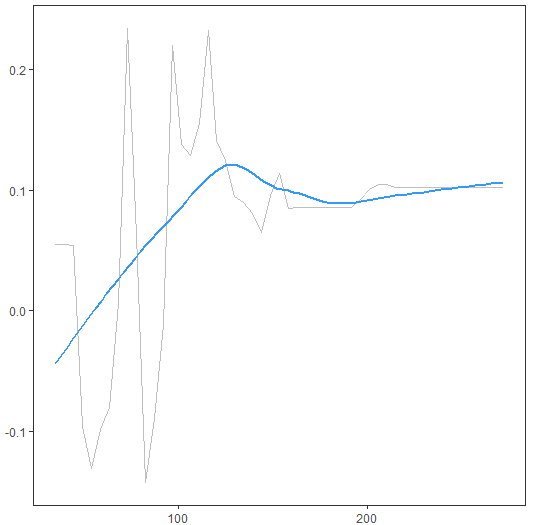

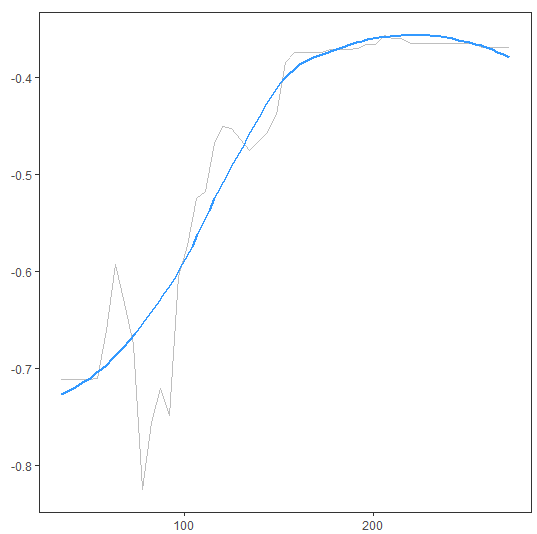

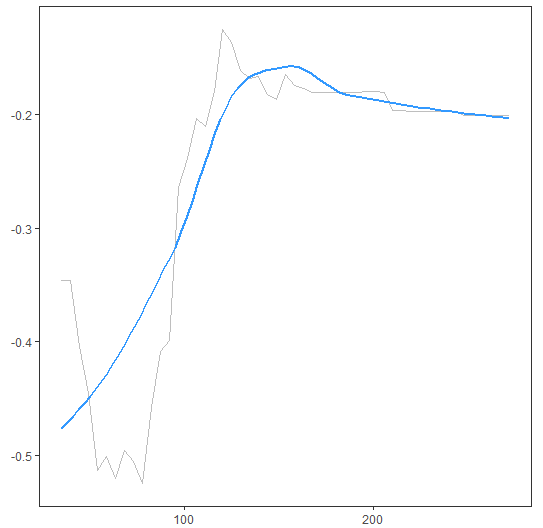


(a)

Partial Dependence


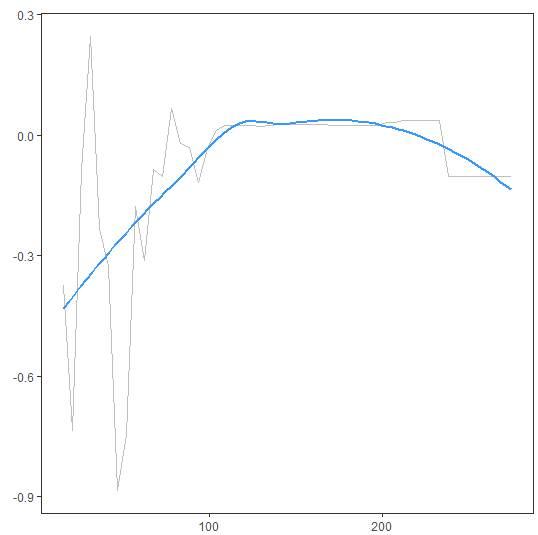

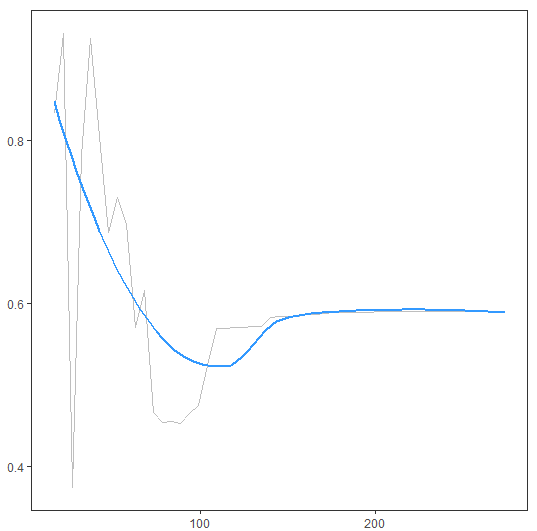

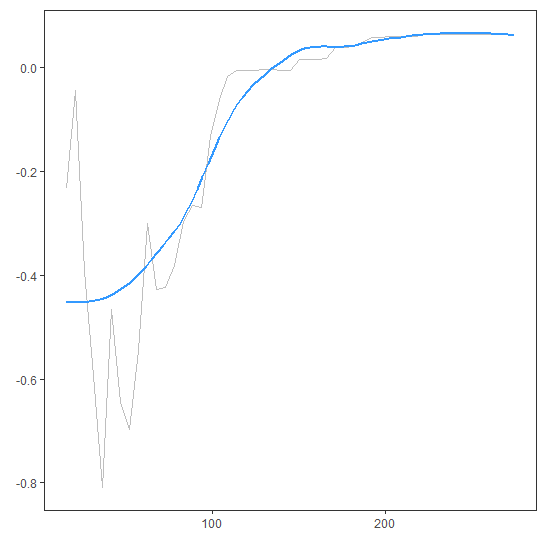

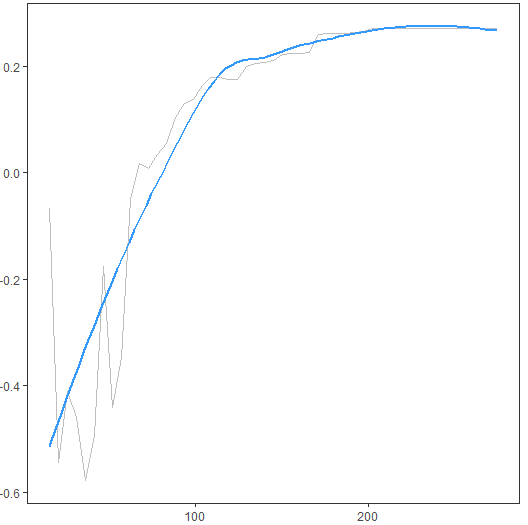


(b)

Length of hospital stay Length of hospital stay Length of hospital stay Length of hospital stay
