# Supplementary material for: Machine learning methods to predict attrition in a population-based cohort of very preterm infants
Source: Sci Rep. 2022 Jun 22;12:10587. doi: 10.1038/s41598-022-13946-z (PMC9217966; doi:10.1038/s41598-022-13946-z)
Supplement: Supplementary file 2 — Supplementary Information 2. [file 41598_2022_13946_MOESM2_ESM.docx]

Supplementary Table 1. Description of predictors taken from baseline and three follow-ups.

| **PREDICTORS** | **DESCRIPTION** |
| --- | --- |
| ***Baseline*** |  |
| Gestational age | Completed weeks – Continuous |
| Type of pregnancy | Singleton/Multiple – Binary |
| Participant's sex | Male/Female – Binary |
| Region of birth | Northern/Lisbon and Tagus Valley – Binary |
| Level of care | Level II - medium intensive / Level III - high-intensive – Binary |
| Birthweight | Grams – Continuous |
| Maternal age | Years – Continuous |
| Native (born in Portugal) | Yes/No – Binary |
| Parity | 0;1; 2; 3 or more – Categorical |
| Type of delivery | Vaginal/ Caesarean – Binary |
| Congenital anomaly | Yes/No – Binary |
| Transfer between NICU | Yes/No – Binary |
| Length of hospital stay | Discrete |
| Breastfeeding at discharge | Yes/No – Binary |
| Level of morbidity | 0 - none/ 1 - at least one/ 2- two or more – Categorical |
| Bronchopulmonary dysplasia (BPD) | Yes/No – Binary |
| Intraventricular haemorrhage (IVH) | Yes/No – Binary |
| Cystic Periventricular Leukomalacia (cPVL) | Yes/No – Binary |
| Late infection confirmed (> 72 hours of life) | Yes/No – Binary |
| Retinopathy of prematurity (ROP) | Yes/No – Binary |
| Necrotizing enterocolitis (NEC) | Yes/No – Binary |
| Treatment for Patent ductus arteriosus (PDA) | Yes/No – Binary |
| Typology of urban area | APU = predominantly urban; AMU = moderately urban; APR = predominantly rural – Categorical |
| European Deprivation Index (Portuguese version)^49^ | 1- least deprived/ 5- most deprived – Quintile score |
| ***Follow-up 1*** |  |
| Family member who answered the questionnaire | Mother/Father/Both parents/Grandparents/Foster care/ Other – Categorical |
| Parents' perceptions of their child's health | 1 -Excellent/ 2- very good/ 3- good/ 4-fair /5- poor – Categorical |
| ***Follow-up 2*** |  |
| Family member who answered the questionnaire | Mother/Father/Both parents/Grandparents/Foster care/ Other – Categorical |
| Hospitalization (> 1 day) since hospital discharge | Yes/No – Binary |
| Number of hospitalizations (> 1 day) since hospital discharge | Discrete |
| Parents' perceptions of their child's growth | Yes/No/ I don’t know – Categorical |
| Current main activity - Mother | Full-time employed/ Part-time employed/ Unemployed / Student /Parental leave /Fulfilling domestic tasks/Home parent – Categorical |
| Mother’s educational level | Primary/ Low secondary /Upper secondary/Post-secondary, not tertiary/ Short cycle tertiary/Bachelor degree/Master degree/Doctoral degree – Categorical |
| Mother's marital Status | Living alone /Living with child's father/ Living with another partner |
| Current main activity - Partner | Full-time employed/ Part-time employed/ Unemployed / Student /Parental leave /Fulfilling domestic tasks/Home parent – Categorical |
| Partner’s educational level | Primary/ Low secondary /Upper secondary/Post-secondary, not tertiary/ Short cycle tertiary/Bachelor degree/Master degree/Doctoral degree – Categorical |
| Number of children in the household (including EPICE's baby) | Discrete |
| Who the child live with | Both parents/Mother alone/Father alone/Alternates between mother and father/Other family member/Care home/ Other – Categorical |
| Nonverbal cognitive development (moderate to severe impairment)^a^ | Yes/No – Binary |
| Severe neurosensory impairment ^a,b^ | Yes/No – Binary |
| Cerebral palsy | Yes/No – Binary |
| Child speak at least 10 words | Yes/No – Binary |
| Child speaks the language of the country or birth | Yes/No – Binary |
| Child was ever breastfed | Yes/No – Binary |
| ***Follow-up 3*** |  |
| Family member who answered the questionnaire | Mother/Father/Both parents/Grandparents/Foster care/ Other- Categorical |
| Need of any regular medical care/assistance - (yes/no) | Yes/No – Binary |
| Hospitalization (> 1 day) since 2 years old | Yes/No – Binary |
| Need of special education - (yes/no) | Yes/No – Binary |
| ^a^ Variables previously described for the EPICE cohort ^55,56.^  ^b^ Children with one or more of severe hearing, vision or gross motor impairment. | |
