# Supplementary material for: Machine learning methods to predict attrition in a population-based cohort of very preterm infants
Source: Sci Rep. 2022 Jun 22;12:10587. doi: 10.1038/s41598-022-13946-z (PMC9217966; doi:10.1038/s41598-022-13946-z)
Supplement: Supplementary file 3 — Supplementary Information 3. [file 41598_2022_13946_MOESM3_ESM.docx]

Supplementary Table 2. Odds-ratios of Logistic Regression in the baseline model.

| **Follow-up** | **Predictors (top-ranked variables)** | **Odds Ratio** |
| --- | --- | --- |
| **1** | Birthweight | 1,00 |
|  | Maternal age | 1,08 |
|  | Length of hospital stay | 0,93 |
|  | Gestational age | 0,77 |
|  | Sex (female) | 1,63 |
| **2** | Birthweight | 1,00 |
|  | Gestational age | 1,01 |
|  | Maternal age | 1,05 |
|  | Length of hospital stay | 0,98 |
|  | Region of birth(Lisbon and Tagus Valley) | 0,68 |
| **3** | Birthweight | 1,00 |
|  | Maternal age | 0,95 |
|  | Gestational age | 1,08 |
|  | Length of hospital stay | 0,98 |
|  | Sex (female) | 2,05 |
| **4** | Birthweight | 1,01 |
|  | Region of birth (Northern) | 0,55 |
|  | Gestational age | 0,94 |
|  | Length of hospital stay | 0,94 |
|  | Maternal age | 1,15 |
